# Transcriptome analysis reveals the molecular mechanism of yield increases in maize under stable soil water supply

**DOI:** 10.1371/journal.pone.0257756

**Published:** 2021-09-24

**Authors:** Jili Zhang, Peng Wang, Jinfeng Ji, Huaiyu Long, Xia Wu

**Affiliations:** 1 Agronomy College, Heilongjiang Bayi Agricultural University, Daqing, Heilongjiang Province, P. R. China; 2 Institute of Agricultural Resources and Regional Planning, Chinese Academy of Agricultural Sciences, Beijing, P. R. China; United Arab Emirates University, UNITED ARAB EMIRATES

## Abstract

This study explored the physiological and molecular mechanisms of yield increase in maize under stable soil water content (SW) conditions. Results of the study showed that under SW conditions, corn yield increased by 38.72 and 44.09% in 2019 and 2020, respectively. Further, it was found that dry matter accumulation, economic coefficient and photosynthetic rate also increased by 31.24 and 25.67%, 5.45 and 15.38% as well as 29.60 and 31.83% in 2019 and 2020 respectively. However, the results showed that both the activity of antioxidant enzymes and content of osmotic adjustment substances decreased in maize under SW conditions. When compared with soil moisture content of dry and wet alternation (DW) conditions, SW could not only significantly promote growth and yield of maize but also increase the economic coefficient. Transcriptome profiles of maize leaves under the two conditions (SW and DW) were also analyzed and compared. It was found that 11 genes were highly up-regulated in the photosynthesis pathway. These genes included photosystem II protein V (*PsbE*), photosystem II protein VI (*PsbF*), photosystem II protein D1 (*PsbA*), photosystem II protein D2 (*PsbD*) and ATP synthase CF1 beta subunit (*atpB*). Further, it was found that four genes were up-regulated in the oxidative phosphorylation pathway., These were ATP synthase CF1 epsilon subunit (*atpE*), ATP synthase CF1 beta subunit (*atpB*), NADH dehydrogenase subunit 4L (*ndhE*) and NADH dehydrogenase subunit 6 (*ndhG*). In conclusion, the physiological mechanism of stable soil water content (SW) to increase corn yield may be the enhancement of photosynthetic capacity and energy metabolism.

## Introduction

Maize (*Zea mays* L.) is a water-intensive crop. It requires soil water content of 70 to 80% for its growth and yield increase [1,2]. Irrigation is a common practice in maize production. During irrigation process, soil water content is high enough at the initial stage and then declines. Therefore, soil water content is in multiple dry-wet alternation cycles which is unfavorable for crop growth and efficiency of water use is much reduced [3].

In the past two decades, negative pressure irrigation (NPI) has been successfully used to control soil water content and achieves high-efficient use of water in production of many crops [4–6]. Stable soil water content (SW) is capable of improving crop growth and yield as well as increase efficiency of water use. For example, previous studies have shown that SW could increase crop leaf area, photosynthetic rate, root activity [7–9], promote the formation and transport of photosynthates [7] and improve the biological yield as well as the final yield in many crops [9–13]. Stable soil water content (SW) also significantly improves nitrate reductase activity in flue-cured tobacco (Nicotiana tabacum L.) [14] and reduces the osmotic regulating substances in eggplant (Solanum melongena L.) [8,10]. Furthermore, it has been reported that stable soil water content (SW) can improve the efficiency of water use by crops and hence saves water [11,12,15].

In this study, with soil water content of “dry-wet” cycle as the control, the effects of stable soil water content on maize physiological characteristics, dry matter accumulation and yield were evaluated. The mechanism of yield increase in maize was also explored using RNA-seq technology. Therefore, this study provides a new perspective for analyzing the relationship between corn yield and soil moisture.

## Materials and methods

### Materials and experiment design

Experiments were carried out in the rainproof shed at the experimental base of Heilongjiang Bayi Agricultural University in 2019 and 2020. Maize, ‘Xianyu335’ variety was potted at a spacing of 50 cm×50 cm. Each pot contained 30 kg dry soil. To the nutrients contained in these soil (Table 1), some more fertilizers were also applied on the soil (N = 6.00 g/pot, P_2_O_5_ = 3.00 g/pot, and K_2_O = 4.50 g/pot).

**Table 1:** Soil nutrient content selected in the experiment.

Year	Soil type	Organic matter (g/kg)	Alkaline Hydrolysis of Nitrogen (mg/kg)	Available phosphorus (mg/kg)	Quick-acting potassium (mg/kg)	pH
2019	Chernozem	43.58	49.67	13.89	164.48	8.67
2020	Chernozem	44.22	192.92	14.08	166.00	8.37

Maize seeds were planted on 23^rd^ May, germinated on 1^st^ June and reached to the three-leaf stage on 9^th^ June. The soil moisture content during this period was kept at 60 to 70% FC (field capacity) to ensure proper growth of plants. Different water treatments were started from the three-leaf stage on 9^th^ June.

There were two treatments in the experiment. One was the control (CK) and named as DW, because the soil moisture content of the treatment was always in a state of dry-wet alternation during water treatment. When the soil moisture content dropped from 100 to 50% field capacity (FC) a Markov bottle would be used to supply water in drip-irrigation manner. A soil water content of 100% FC would be achieved in two hours. The second treatment was named as SW, where the soil water content at 5–40 cm soil depth was maintained at about 80% FC (within an error of 2%). A soil moisture stabilization controller continuously stabilize the water supply [2]. Soil moisture content in both treatments was monitored daily at 18:00 using a soil moisture analyzer (Beijing ruixinlong electronic technology research institute, China). Each treatment contained 27 plants and the 2019 and 2020 trials were handled similarly.

The experiments were set in triplicates for every analysis index with 27 plants in each experiment and one plant per pot. These cultivation pots were placed in a 50 cm×50 cm grid and with each grid 50 cm apart.

### Sample collection and evaluated indicators

#### Determination of physiological indicators

This study used the five key periods of corn growth. These were: 31^st^ day (joint period), 47^th^ days (big bell mouth period), 63^rd^ days (tasseling period), 97^th^ days (filling period) and 123^rd^ days (mature period) after water treatment. The chlorophyll a, chlorophyll b and carotenoid contents, nitrate reductase and antioxidant enzyme (POD, SOD and CAT) activity as well as the content of three osmotic adjustment substances (free proline, soluble protein content and soluble sugar content) were evaluated at the 31, 47, 63, 97 and 123 day after water treatment. Three plants per treatment were measured and averaged. At the five periods the ear leaves (the 9th and 10th leaf) were chosen as the sample because they have a greater impact on the yield. Two leaves were chosen because ‘Xianyu335’ variety has two ears.

The 95% ethanol extraction method was used to determine chlorophyll *a*, chlorophyll *b*, and carotenoids content [13]. Nitrate reductase activity was measured by the aminobenzene sulfonic acid-based colorimetric method was employed to measure. The NBT reduction method, guaiacol colorimetric method and ultraviolet colorimetric method were used for measuring superoxide dismutase (SOD), peroxidase isozyme (POD) and Catalase enzymes (CAT) activity, respectively [16]. The soluble protein, soluble sugar, and free proline contents were determined through coomassie brilliant blue, anthrone colorimetry and ninhydrin methods, respectively [16].

In addition, the net photosynthetic rate, transpiration rate, intercellular CO_2_ concentration and stomatal conductance were determined using LI-6400 portable photosynthetic apparatus. This was performed by measuring the 9^th^ and 10^th^ leaf at 10:00 am on the 31^st^ day after treatment [15]. Three plants were measured per treatment and the values were averaged.

#### Determination of dry matter accumulation, yield, economic coefficient, and economic yield water use efficiency

Dry matter accumulation was measured at the 31, 47, 63, 97 and 123 day after water treatment, three plants were randomly collected from each treatment and dried to a constant weight at 70°C.

Corn matured on the 123 day of water treatment and then kernels from three plants were harvested and the yield per plant (at water content of 14%) was achieved. Then economic coefficient was also calculated.

Economic coefficient (%) = Economic output (g)/Biological output (g)

The total irrigation water amount of each treatment was summed up and economic yield water use efficiency (EWUE) was achieved according to the following equation:

EWUE (g/kg) = Total grain weight of each treatment (g)/Water consumption amount (kg)

### Transcriptome sequencing

The leaf tissues collected after 31st day of water treatment were immediately placed in liquid nitrogen. The leaves were then transferred to -80°C freezer for temporary storage, then used for transcriptome sequencing.

#### Massager- RNA (mRNA) library construction and sequencing

Total RNA was isolated and purified using TRIzol reagent (Invitrogen, Carlsbad, CA, USA) following procedure provided by the manufacturer. The RNA amount and purity of each sample was quantified using NanoDrop ND-1000 (NanoDrop, Wilmington, DE, USA). The RNA integrity was assessed by Bioanalyzer 2100 (Agilent, CA, USA) with RIN number >7.0, and confirmed by electrophoresis with denaturing agarose gel. Poly (A) RNA is purified from 1μg total RNA using Dynabeads Oligo (dT) 25–61005 (Thermo Fisher, CA, USA) using two rounds of purification. Then the poly (A) RNA was fragmented into small pieces using Magnesium RNA Fragmentation Module (NEB, cat.e6150, USA) under 94°C 5-7min. Then the cleaved RNA fragments were reverse-transcribed to create the cDNAs by SuperScript™ II Reverse Transcriptase (Invitrogen, cat. 1896649, USA) which were later used to synthesize U-labeled second-stranded DNAs with E. coli DNA polymerase I (NEB, cat.m0209, USA), RNase H (NEB, cat.m0297, USA) and dUTP Solution (Thermo Fisher, cat.R0133, USA. An A-base was then added to the blunt ends of each strand, preparing them for ligation to the indexed adapters. Each adapter contained a T-base overhang for ligating the adapter to the A-tailed fragmented DNA. Single- or dual-index adapters were ligated to the fragments and size selection was performed with AMPureXP beads. After the heat-labile UDG enzyme (NEB, cat.m0280, USA) treatment of the U-labeled second-stranded DNAs, the ligated products are amplified with PCR through the following conditions: initial denaturation at 95°C for 3 min; 8 cycles of denaturation at 98°C for 15 sec, annealing at 60°C for 15 sec and extension at 72°C for 30 sec; and then final extension at 72°C for 5 min. The average insert size for the final cDNA library was 300±50 bp. The 2×150bp paired-end sequencing (PE150) was subsequently performed on an Illumina Novaseq™ 6000 (LC-Bio Technology CO., Ltd., Hangzhou, China) following the vendor’s recommended protocol.

#### Sequence and primary analysis

Cutadapt software (https://cutadapt.readthedocs.io/en/stable/, version: cutadapt-1.9) was used to remove the reads that contained adaptor contamination, (ADAPT1: 5’-AATGATACGGCGACCACCGAGATCTACACTCTTTCCCTACACGACGCTCTTCCGATCT-3’; ADAPT2: 5’-GATCGGAAGAGCACACGTCTGAACTCCAGTCAC(index)ATCTCGTATGCCGTCTTCTGCTTG-3’). After the low quality bases and undetermined bases were removed, HISAT2 software (https://daehwankimlab.github.io/hisat2/, version: hisat2-2.0.4) was used to map reads to the genome (command line: ~hisat2–1 R1.fastq.gz -2 R1.fastq.gz -S sample_mapped.sam). The mapped reads of each sample were assembled using StringTie (http://ccb.jhu.edu/software/stringtie/, version: stringtie-1.3.4d.Linux_x86_64) with default parameters (command line: ~stringtie -p 4 -G genome.gtf -o output.gtf -l sample input.bam). The individual assemblies were merged to get a comprehensive transcriptome using gffcompare software (http://ccb.jhu.edu/software/stringtie/gffcompare.shtml, version: gffcompare-0.9.8.Linux_x86_64) containing 137278 transcripts with an N50 value of 3629 bp. After the final transcriptome was generated, StringTie and ballgown (http://www.bioconductor.org/packages/release/bioc/html/ballgown.html) were used to estimate the expression levels of all transcripts and perform expression level for mRNAs by calculating FPKM (FPKM = [total_exon_fragments/mapped reads(millions) × exon length(kB)]), (command line: ~stringtie -e -B -p 4 -G merged.gtf -o samples.gtf samples.bam). The differentially expressed mRNAs were selected with fold change > 2 or fold change < 0.5 and p value < 0.05 by R package edgeR (https://bioconductor.org/packages/release/bioc/html/edgeR.html) or DESeq2 (http://www.bioconductor.org/packages/release/bioc/html/DESeq2.html), and then analysis GO enrichment and KEGG enrichment to the differentially expressed mRNAs.

### Quantitative real-time PCR validation

Validation of RNA-seq results was performed for 6 randomly selected genes using quantitative RT-PCR. The verification test was commissioned to Beijing Biomarker Biotechnology Co., Ltd to completion. The instruments used in the experiment included analytikjena-qTOWER2.2 fluorescence quantitative PCR instrument (Germany), SCILOGEX D3024R centrifuge (US), ordinary PCR instrument analytikjena-Easycycler (Germany), ultra-micro nucleic acid protein analyzer scandrop100 (Germany) and pipette (American bio-rad). The cDNA of RNA was synthesized using the Aidlab’s reverse transcription kit (TUREscript 1st Stand cDNA SYNTHESIS Kit) for reverse transcription, using a 20-μL reaction system. The primer information was as shown in S3 Table.

### Data analysis

All the collected data in this study were analyzed using IBM SPSS Statistics ver. 18 (SPSS Inc., IL, USA). The Duncan’s Multiple Range tests (DMRT) and post-hoc tests were conducted to compare means between the treatments. The significant difference level was set to P<0.05.

## Results

### Soil moisture

**Fig 1:**
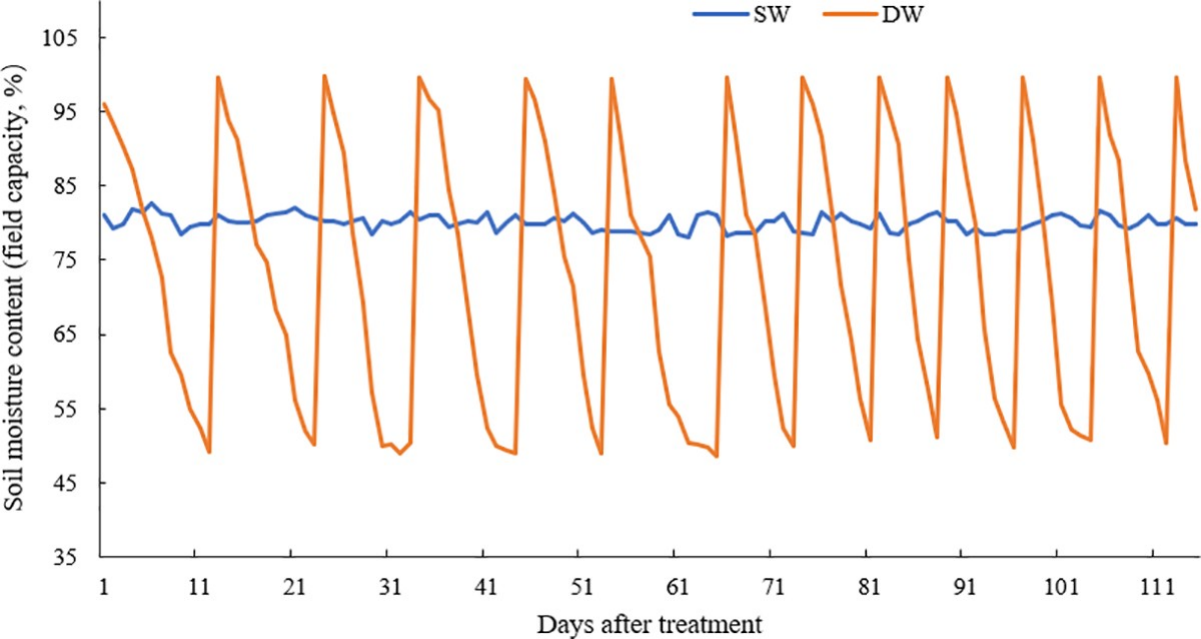
Soil water percentage in field capacity under different treatments. SW is the treatment for stable soil moisture content, and DW is the alternate dry and wet treatment.

Soil moisture content in the two treatments were as presented in Fig 1. Soil water content in DW treatment ranged between 50 and 100% FC during treatment period, with the amplitude of variation of 50.00% FC. The soil moisture of DW experiment set up underwent dry and wet alternations, and a dry-wet alternate cycle averaged at 10.83 d in the early 65 days after different irrigation treatment, and averaged 7.83 d from the 66 to 123 day of treatment. In SW treatment, soil water content was basically stable and maintained at between 78.51 and- 82.00% FC from the onset of treatment to the end of the experiment.

### Chlorophyll *a*, *b*, and carotenoid content, and photosynthesis characteristics

In the two years, the content of chlorophyll *a*, *b* in SW treatment was significantly lower than that in DW treatment from 31 to 63 day after water treatment (Table 2). The content of carotenoids in SW was also significantly lower than that in DW treatment from 31 to 63 days and 31 to 47 days after water treatment in 2019 and 2020, respectively. The results indicated that the contents of chlorophyll and carotenoids in corn leaves were significantly influenced by stable soil moisture content.

**Table 2:** The pigment content, nitrate reductase activity, antioxidant enzyme activity, osmotic regulating substance content and dry matter accumulation.

Item	Treatment	31 day		47 day		63 day		97 day		123 day	
		2019	2020	2019	2020	2019	2020	2019	2020	2019	2020
Chlorophyll *a* (mg/g)	SW^1)^	2.18±0.04b^2)^	1.30±0.06b	2.02±0.09b	1.38±0.04b	0.34±0.01b	1.25±0.07b	0.89±0.03a	1.17±0.05b	0.36±0.04a	0.99±0.16a
	DW	2.97±0.10a	1.55±0.06a	2.67±0.05a	1.71±0.03a	0.41±0.01a	1.43±0.05a	1.05±0.18a	1.47±0.01a	0.41±0.01a	1.06±0.17a
Chlorophyll *b* (mg/g)	SW	0.55±0.04b	0.36±0.04b	0.54±0.06b	0.39±0.02b	0.93±0.08b	0.35±0.02b	0.27±0.02a	0.33±0.07a	0.09±0.01b	0.25±0.04b
	DW	0.84±0.03a	0.44±0.03a	0.79±0.03a	0.46±0.02a	1.19±0.03a	0.38±0.04a	0.23±0.02a	0.38±0.04a	0.12±0.01a	0.30±0.04a
Carotenoids (mg/g)	SW	1.08±0.06b	0.55±0.01b	0.95±0.03b	0.58±0.02b	0.87±0.07b	0.63±0.03a	0.49±0.04a	0.56±0.04b	0.24±0.03b	0.43±0.03a
	DW	1.31±0.06a	0.65±0.01a	1.26±0.04a	0.66±0.03a	1.05±0.06a	0.60±0.05a	0.52±0.04a	0.69±0.06a	0.36±0.04a	0.46±0.03a
Nitrate reductase activity (μg/(g/h))	SW	145.14±6.42a	116.33±11.63a	16.28±2.16a	268.18±0.68a	251.40±22.38a	362.98±0.26a	33.37±0.30a	185.70±4.44a	38.00±5.08a	51.55±1.89a
	DW	85.88±7.31b	55.69±0.79b	6.66±1.04b	232.90±5.15b	170.09±11.74b	354.55±4.69a	16.29±1.51b	153.75±1.70b	9.57±1.25b	37.02±2.68b
SOD activity (U/g)	SW	115.56±1.41b	135.68±5.70a	108.25±11.14b	244.21±5.85b	326.50±2.84b	232.88±7.37b	296.63±40.67b	347.49±11.70b	332.33±35.98b	815.65±25.30b
	DW	53.23±13.56b	101.77±16.78a	151.09±10.69a	279.08±7.78a	375.71±5.47a	249.96±3.66a	390.29±21.46a	467.22±17.79a	401.95±26.20a	941.85±30.47a
POD activity(U/g)	SW	72.31±3.45b	10.99±1.60b	61.11±1.39b	21.31±1.41b	23.35±4.84a	28.47±2.18b	14.69±2.11a	9.76±0.70a	1.86±0.93b	67.65±8.40b
	DW	83.82±3.26a	18.18±2.26a	131.97±4.70a	28.89±1.26a	26.26±4.01a	39.77±5.47a	16.08±0.09a	10.69±2.93a	3.01±1.05a	90.52±11.87a
CAT activity (U/g)	SW	141.23±18.32b	172.00±5.53b	157.41±15.64b	251.74±7.17b	282.63±10.21b	321.22±5.19b	344.49±14.91b	183.28±15.86b	224.66±4.72b	611.19±65.95b
	DW	226.93±13.58a	209.12±15.96a	236.40±10.92a	299.68±15.93a	311.05±14.59a	456.83±28.55a	386.43±4.14a	256.64±6.75a	265.98±6.40a	871.39±42.22a
Free proline (μg/g)	SW	18.81±1.38b	54.27±7.71b	21.56±3.61b	23.18±2.11b	22.46±0.63b	10.72±1.11b	25.89±5.73b	27.40±2.01b	26.39±2.13b	43.34±0.68a
	DW	64.21±4.83a	151.08±22.52a	101.69±3.65a	140.85±7.90a	48.08±2.09a	50.01±13.04a	35.78±5.73a	37.43±2.97a	45.79±3.77a	45.99±2.55a
Soluble protein (mg/g)	SW	4.84±0.35b	17.75±0.97b	7.39±0.19b	17.71±0.49a	13.15±0.31b	20.37±0.63a	15.75±0.85a	22.01±1.02a	13.01±0.37b	15.39±0.47b
	DW	6.43±0.38a	21.48±0.33a	8.44±0.48a	20.47±1.20a	15.93±0.81a	19.89±2.10a	17.52±0.42a	21.23±1.01a	17.82±0.79a	22.83±0.53a
Soluble sugar content (%)	SW	1.17±0.04b	0.95±0.03b	0.94±0.08a	1.33±0.03b	1.54±0.01a	2.74±0.15b	1.17±0.05a	2.53±0.08b	1.56±0.07b	3.37±0.31b
	DW	1.39±0.02a	1.25±0.03a	0.97±0.06a	1.56±0.07a	1.31±0.13a	3.16±0.15a	1.53±0.10a	3.44±0.19a	1.80±0.09a	4.28±0.46a
Dry matter accumulation (g/plant)	SW	19.60±0.97a	76.66±2.14a	46.44±1.86a	101.21±7.19a	61.90±2.03a	164.72±1.90a	-	295.60±13.66a	202.35±6.11a	358.11±18.73a
	DW	15.91±1.30b	69.98±1.97b	35.86±1.95b	99.37±0.98a	52.20±2.84b	150.85±2.99b	-	280.78±6.67a	144.66±0.31b	284.97±9.99b

^1)^ SW is the treatment for stable soil moisture content, and DW is the alternate dry and wet treatment.

^2)^The values in the table are mean±standard deviation; different lowercase letters indicate significant difference at *P*<0.05 level.

The net photosynthetic rate in SW was 29.77% and 31.83% more than in DW treatment in 2019 and 2020 (Table 3), respectively. The net photosynthetic rate between the two treatments were found to be significantly different (P<0.05). The transpiration rate and stomatal conductance of SW treatment were 11.23 and 27.78% more than that of DW, respectively in 2020. The intercellular CO_2_ concentration of SW treatment was 12.22% lower than that in DW treatment in 2020 and the difference between two treatments was significant (P<0.05).

**Table 3:** Effects of different treatments on maize photosynthesis.

Year	Treatment^1)^	Net photosynthetic rate μmol/(m^2^/s)	Transpiration rate mmol/(m^2^/s)	Intercellular CO_2_ concentration μmol/mol	Stomatal conductance mol/(m^2^/s)
2019	SW^1)^	22.20±0.53a^2)^	0.63±0.08a	282.00±12.12b	0.66±0.01a
	DW	17.13±0.67b	0.37±0.02b	368.33±3.51a	0.12±0.01b
2020	SW	32.10±1.75a	5.15±0.31a	129.33±1.53b	0.23±0.01a
	DW	24.35±1.85b	4.63±0.27b	147.33±2.08a	0.18±0.01b

^1)^ SW is the treatment for stable soil moisture content, and DW is the alternate dry and wet treatment.

^2)^The values in the table are mean±standard deviation; different lowercase letters indicate significant difference at *P*<0.05 level.

### Nitrate reductase and antioxidant enzyme activity

Comparing the two treatments, the nitrate reductase activity in maize leaves under SW conditions was higher than that in the leaves under DW conditions of 2019 and 2020. In both years, leaf SOD and CAT activities in SW treatment was significantly lower than that in DW treatment (Table 2); leaf POD activity in SW treatment was significantly lower than in DW treatment from 31 to 47 day after water treatment in 2019 and from 31 to 63 day after treatment in 2020 (P<0.05). This indicates that leaf SOD, POD and CAT activities are affected by stable soil moisture content.

### Free proline, soluble protein, and soluble sugar contents

Both free proline and soluble sugar contents in the leaves of maize under SW were significantly lower than in the leaves of maize under DW in 2019 and 2020 (Table 2). The soluble protein content in the leaves of maize under SW was significantly lower than that of DW at the 31day after water treatment (P<0.05). However, the soluble protein content it was similar in the leaves of maize under the two treatments at 47 and 63 day after water treatment.

### Dry matter accumulation, yield, economic coefficient, and EWUE

Dry matter accumulation and yield were both significantly higher in the maize under SW treatment than that under DW treatment (P<0.05; Table 4). The dry matter accumulation in the maize under SW treatment was 28.51 and 25.67% more than that under DW in 2019 and 2020, respectively. Tthe yield of maize under SW treatment was 38.74 and 44.10% more than that of maize under DW in 2019 and 2020, respectively. The economic coefficient of maize under SW was 7.84 and 13.46% more than that of maize under DW treatment in 2019 and 2020, respectively. The economic coefficient of maize under DW and SW were significantly different (P<0.05). Economic yield water use efficiency (EWUE) of maize under SW treatment were significantly higher than that of maize under DW treatment.

**Table 4:** Yield, economic coefficient and water use efficiency.

Treatment^1)^	Yield (g/plant)		Economic coefficient		EWUE (g/kg)^2)^	
	2019	2020	2019	2020	2019	2020
SW^1)^	111.21±6.14a^2)^	213.57±27.75a	0.55±0.01a	0.59±0.05a	4.37±0.23a	4.68±0.52a
DW	80.16±1.68b	148.21±9.58b	0.51±0.01b	0.52±0.03b	2.35±0.05b	1.35±0.08b

^1)^ SW is the treatment for stable soil moisture content, and DW is the alternate dry and wet treatment.

^2)^ EWUE, economic yield water use efficiency.

### Transcriptome analysis

#### Transcriptome sequencing quality analysis

The illuminahiseq4000 was used to construct maize reference transcriptome (Table 5). A total of 54674630, 46062032 and 51458738 original reads were obtained from the three repeats of maize under SW treatment. On the other hand, a total of 53865048, 60991256 and 50960320 original reads were obtained from the three repeats of maize under DW treatment. After removing the sequencing adapters and low-quality sequencing data, 51699400, 40695806 and 47516998 valid reads (>88.35%) were obtained from the three repeats in maize under SW treatment whereas 53497300, 50110776 and 50655416 valid reads (>82.16%) were obtained from the three repeats in maize under DW treatment were remained for the subsequent assembly. It was found that the valid Ratio was from 88.35% to 94.56% in maize under SW treatment, and from 82.16% to 94.40% in maize under DW treatment. Further, the results show that all treatments were greater than 99.96%, when the sequencing error rate of Q20 value was less than 0.01. On the other hand, when the sequencing error rate of Q30 value was less than 0.001, all samples were greater than 96.90%. It was found that the GC percentages exceeded 50% in all samples. The results of this study indicate that the transcriptome sequencing were of high quantity and quality, which ensures accuracy and reliability of the results. On the basis of comparisons of six randomly selected genes from maize under the SW and DW treatments, the quantitative real-time PCR (RT-PCR) results showed consonance with the RNA sequencing (RNA-seq) data (S5 Fig).

**Table 5:** Summary statistics of the sequencing results.

Sample^1)^	Raw data ^2)^	Valid data ^3)^	Valid ratio(reads) ^3)^	Q20% ^3)^	Q30% ^3)^	GC content (%)
SW-1	54,674,630	51,699,400	94.56	99.96	96.90	50.00
SW-2	46,062,032	40,695,806	88.35	99.97	96.77	50.50
SW-3	51,458,738	47,516,998	92.34	99.98	97.63	51.50
DW-1	53,865,048	53,497,300	99.32	99.98	98.34	52.00
DW-2	60,991,256	50,110,776	82.16	99.97	98.23	51.50
DW-3	50,960,320	50,655,416	99.40	99.98	98.43	53.00

^1)^ SW is the treatment for stable soil moisture content, and DW is the alternate dry and wet treatment, the number after that indicates the measurement sample number.

^2)^ Raw data is the total number of reads of offline data.

^3)^ Valid data is the number of valid reads after removing joints, removing low quality, etc., Valid ratio is the percentage of valid reads, and Q20% is the percentage of bases with a quality value ≥ 20 (Sequencing error rate is less than 0.01), Q30% is the proportion of bases with quality value ≥30 (sequencing error rate is less than 0.001).

#### PCA analysis and differentially expressed genes

Principal component analysis (PCA) was performed to evaluate the differentially expressed genes between the maize under the two treatments (S1A Fig). It was found that the contribution rate of the first principal component to gene difference was 91.67%, the second principal component was 4.97% and the cumulative contribution rate was 96.64%. The difference in soil water content change pattern constituted the main source of the difference in gene expression. Compared with maize under DW conditions, the maize under SW conditions had 1165 differential genes up-regulated and 2232 differential genes down-regulated (S1B Fig).

**Fig 2:**
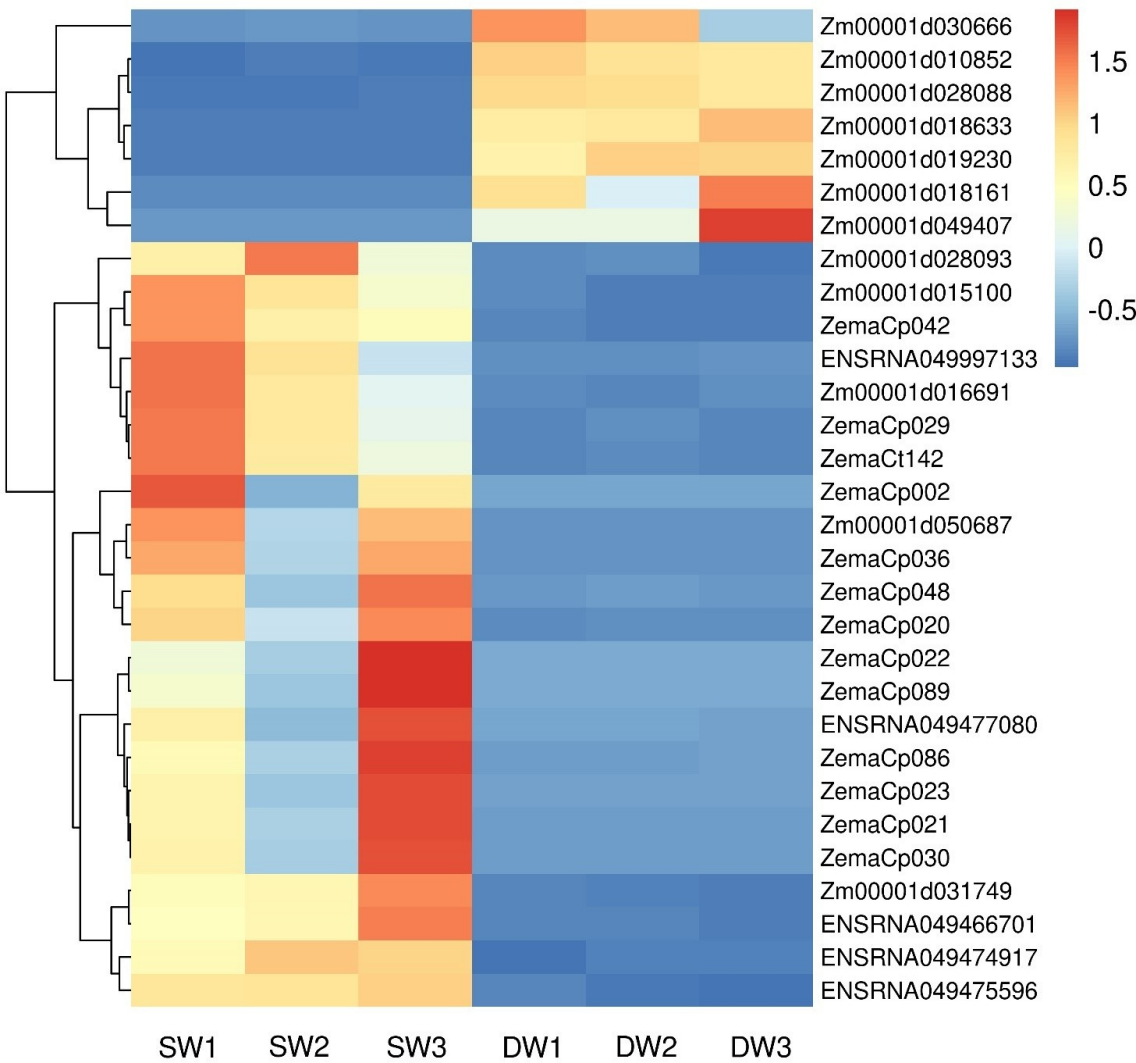
Clustering map of differential genes in maize leaves. SW refers to stable soil moisture content treatment, DW refers to alternately dry and wet treatment, and the number after that indicates the sample number. The abscissa is the sample, the ordinate is the differentially expressed genes screened out, and different colors indicate different gene expression levels, red indicates highly expressed genes, and blue indicates low expressed genes.

Clustering the differentially expressed genes of different water treatments can cluster the genes with the same or similar expression into one group. This is to enable them analyze the unknown function of the known gene or the prediction of the unknown gene function. In this study, the FPKM value of gene expression was used to cluster the differentially expressed genes between the plants under the two soil water treatments (Fig 2). The top 30 genes of Log_10_ (FPKM) among all differential genes were analyzed from large to small (Fig 2). Specifically, there were seven highly down-regulated genes, which were divided into three categories of cellular component, biological process, and molecular function, including 39 GO terms such as oxidoreductase activity, metal ion binding, protein binding and protein targeting to chloroplast, and so on. The other 23 genes were up-regulated and divided into three categories: cellular component, biological process and molecular function. These includes the 97 GO terms such as chloroplast thylakoid membrane, photosynthesis, chloroplast, ATP binding, plastid and ATP biosynthetic process. The 23 up-regulated genes are mainly distributed in eight KEGG pathways, including ribosome, plant-pathogen interaction, photosynthesis, oxidative phosphorylation and beta-alanine metabolism.

#### Functional classification of differentially expressed genes

Compared with the genes in maize under DW, a total of 1,936 significant differential genes are distributed in 267 GO terms in maize under SW treatment. These were divided into three categories: biological process, cellular component and molecular function (S2 Fig). The biological process mainly includes GO terms such as oxidation-reduction process, phosphorylation and protein autophosphorylation. On the other hand, the cellular components includes GO terms such as membrane, integral component of plasma membrane, nucleus, plastid and chloroplast while molecular functions includes GO terms such as protein binding, ATP binding and transferase activity.

#### GO enrichment analysis of differentially expressed genes

**Fig 3:**
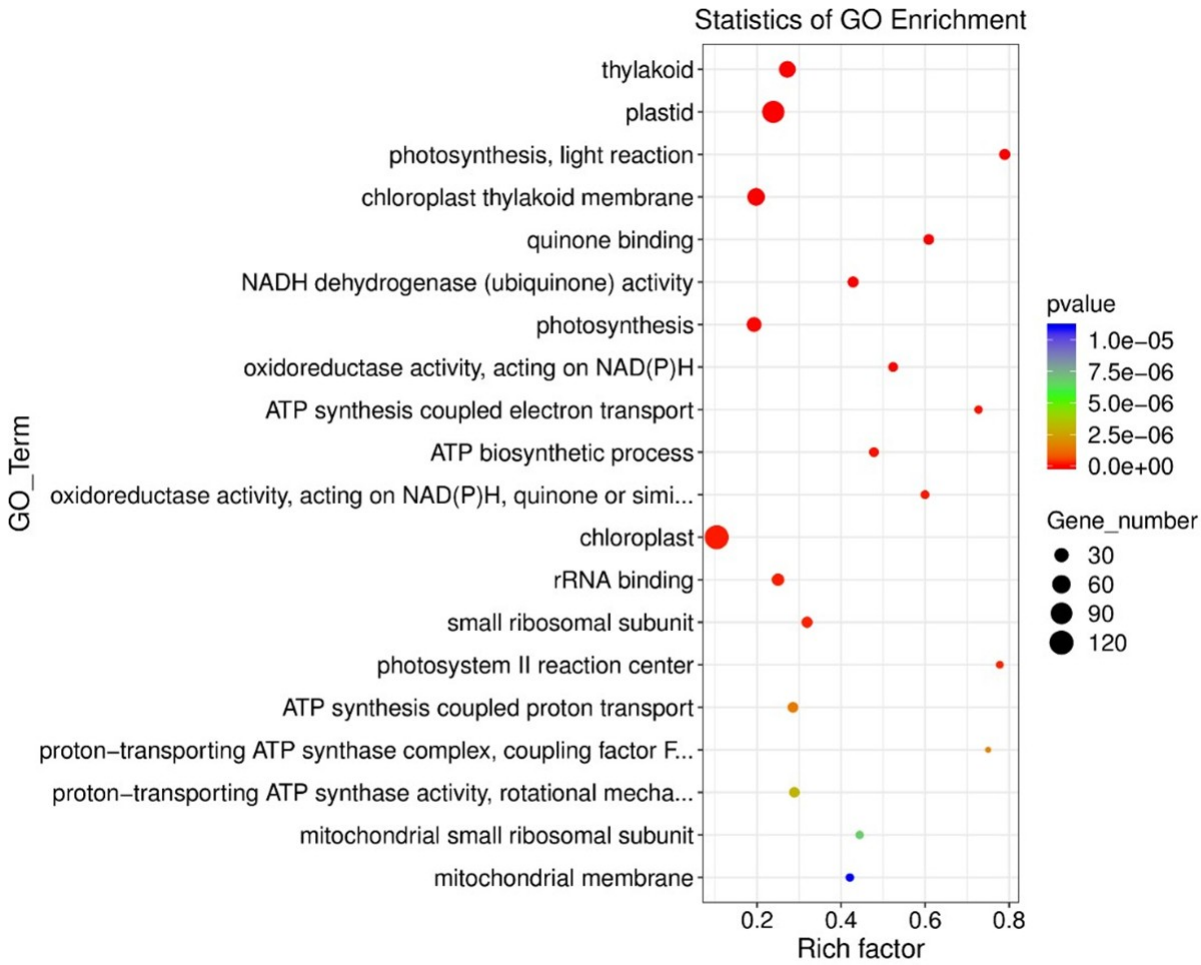
The GO enrichment analysis of the differentially expressed genes in maize. The horizontal axis represents the degree of enrichment (rich factor), the vertical axis represents the enriched GO term; the size of the dot represents the number of differential genes enriched in a GO term; the color of the dot represents the different *p* value; rich factor represents the number of differential genes belonging to a GO term/total number of genes belonging to this GO. The higher rich factor is, the higher the enrichment of GO.

In this study an R script was written according to the hypergeometric test formula used for GO analysis. Top 20 GO terms with the smallest P value in the enrichment analysis results were selected to draw a scatter plot (Fig 3). Chloroplast, plastid, thylakoid and chloroplast thylakoid membrane were the four GO terms that were significantly different and each has the large number of enriched genes. There were 120 different genes in chloroplast, of which 82 genes were up-regulated and38 genes were down-regulated. Plastid had a total of 96 differential genes, of which 76 genes were up-regulated and20 genes were down-regulated. There are 46 differential genes in thylakoid, of which 43 genes are up-regulated, and 3 (*H4C7*, *Zm00001d026523* and *Zm00001d032051*) genes are down-regulated. There were 46 differential genes in thylakoid, of which 43 genes were up-regulated and 3 (*H4C7*, *Zm00001d026523* and *Zm00001d032051*) genes were down-regulated. There are 53 differential genes in chloroplast thylakoid membrane, of which 45 genes are up-regulated and 8 genes (*Zm00001d015095* (harpin binding protein1), *Zm00001d016826* (Chlorophyll A-B binding family protein), *Zm00001d018763*, *Zm00001d026523*, *Zm00001d028256* (proline-rich family protein), *Zm00001d028756*, *Zm00001d031969* (RNA-binding protein CP31B chloroplastic), *Zm00001d032051* (light harvesting complex photosystem II subunit 6) were down-regulated.

Photosynthesis, light reaction and photosystem II reaction center were highly enriched. These results indicated that under stable soil water content, the number and degree of enrichment of GO term significant differential genes related to photosynthesis in maize leaves were relatively high (P<0.05).

#### KEGG function enrichment analysis

**Fig 4:**
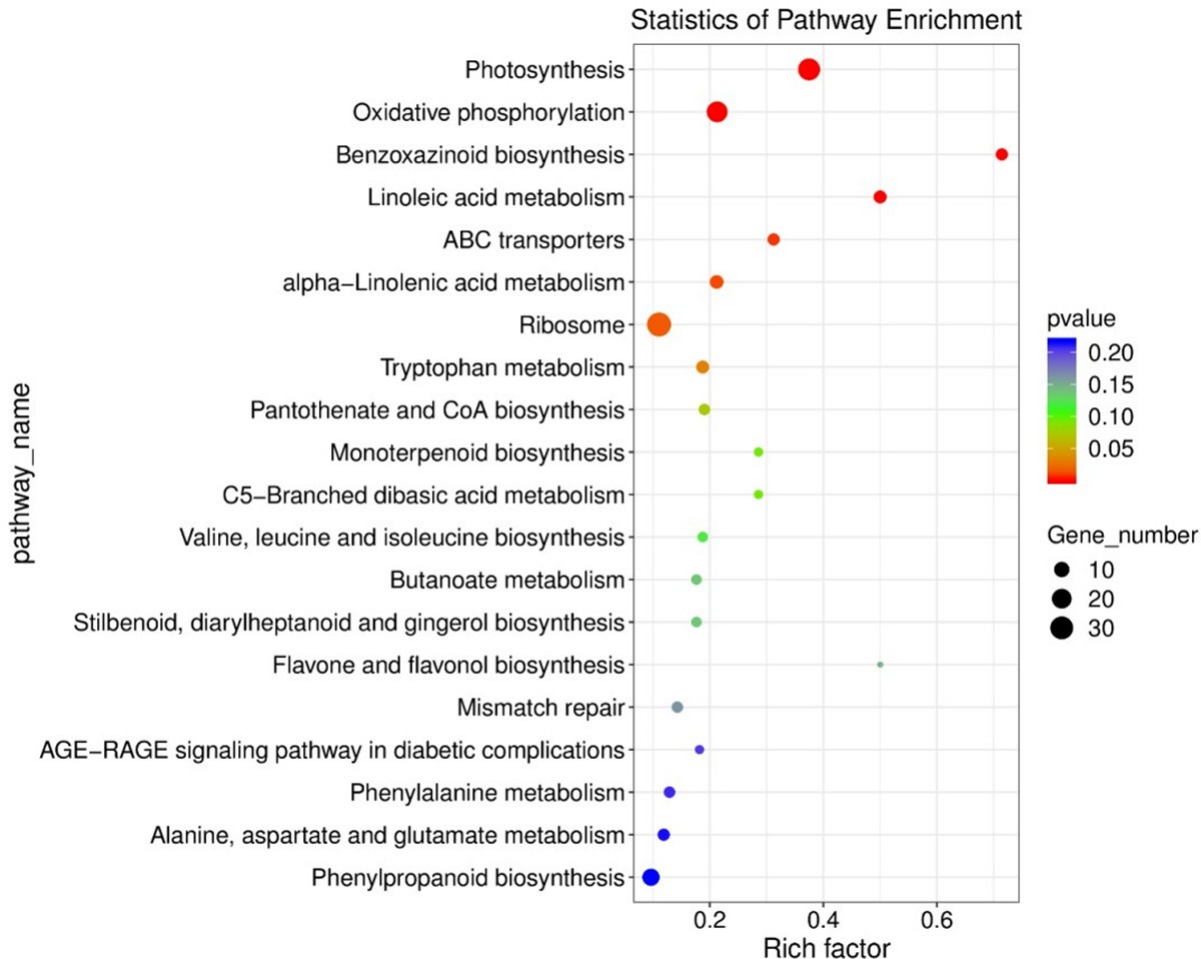
Enrichment analysis of the differentially expressed gene at KEGG in maize. The horizontal axis indicates the degree of enrichment (Rich factor), and the vertical axis indicates the enriched KEGG pathway; the size of the dot indicates the number of differential genes enriched in a certain KEGG pathway; the color of the dot indicates *p* values; Rich Factor represents the number of differential genes belonging to a certain KEGG pathway/total number of genes belonging to this KEGG pathway. The higher the Rich factor, the higher the enrichment of the KEGG pathway.

KEGG enrichment analysis of differentially expressed genes under different irrigation treatments can find significantly enriched metabolic pathways or signal transduction pathways. In this study, the top 20 pathways with the smallest P-value were used to draw a scatter diagram (Fig 4). Compared with the maize under DW conditions, the maize under SW had a larger number of pathway-enriched genes in photosynthesis, oxidative phosphorylation and ribosome pathway. The highly enriched genes were in the two metabolic pathway genes of benzoxazinoid biosynthesis and linoleic acid metabolism. There were a total of 27 significantly different genes in the photosynthesis pathway, of which 24 genes were up-regulated, and only 3 genes (*photosystem II oxygen evolving polypeptide2*, *ferredoxin1* and *Zm00001d049732*) were down-regulated. Twenty genes were enriched in the oxidative phosphorylation pathway, of which 16 genes were up-regulated and 4 were down-regulated. A total of 34 genes were enriched in the ribosome pathway, 18 of which were up-regulated and 16 genes were down-regulated.

#### Differentially expressed genes involved in photosynthesis and oxidative phosphorylation pathways

Through analyzing the enrichment of GO genes differentially expressed in maize leaves, it was found that there were more differentially expressed genes in the functional categories of thylakoid, plastid, chloroplast thylakoid membrane, photosynthesis and light reaction whereas ATP synthesis coupled electron transport were also highly enriched. Through KEGG enrichment analysis, only two metabolic pathways, photosynthesis and oxidative phosphorylation, enriched over 20 significant differential genes with Rich factor >0.2, indicating that these two pathways responds to stable soil moisture content in maize and can improve plant physiological characteristics as well as the final yield of maize.

**Fig 5:**
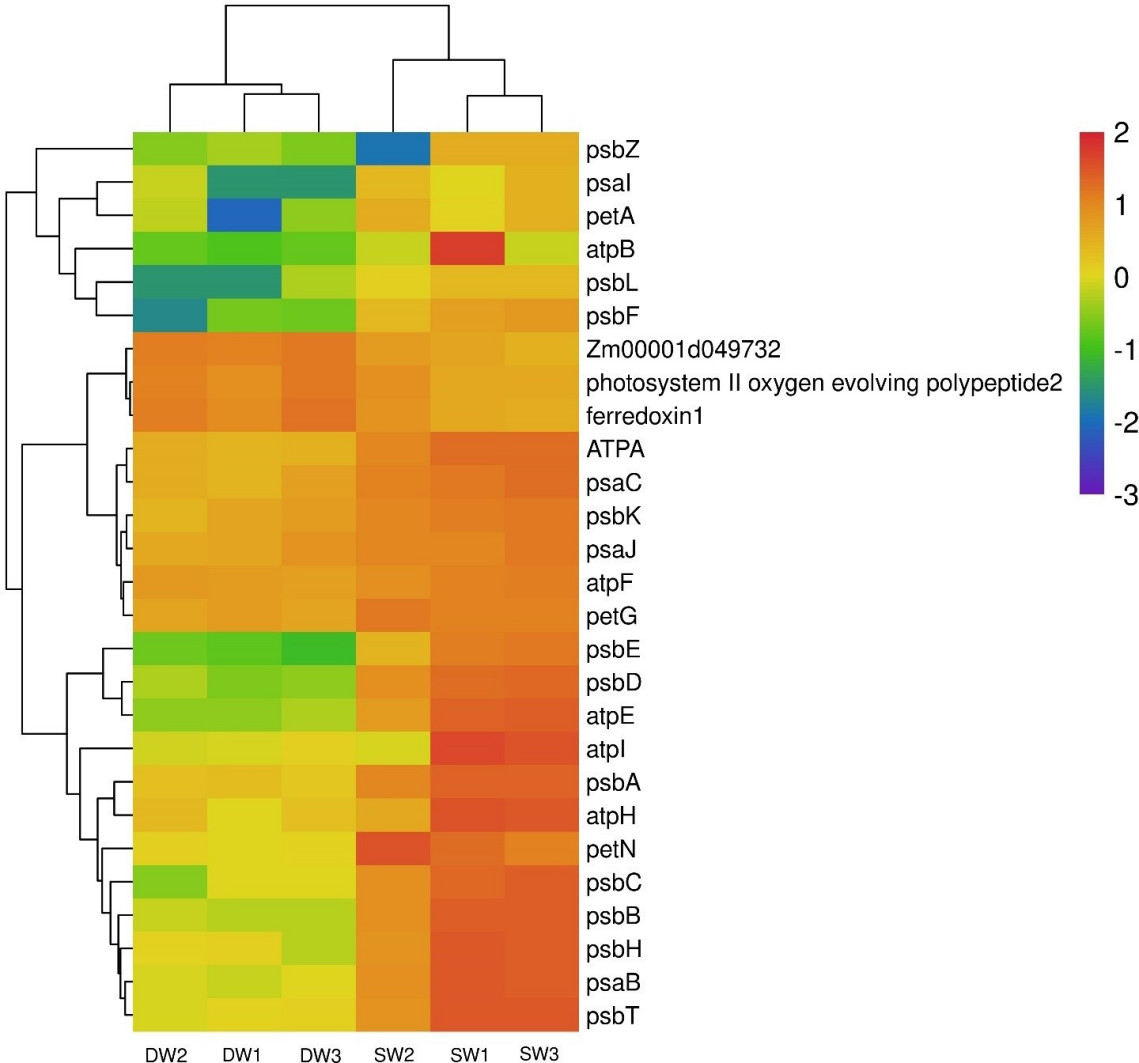
Heat map of photosynthesis pathway gene expression. SW refers to stable soil moisture content treatment, DW refers to alternately dry and wet treatment, and the number after that indicates the sample number.

**Fig 6:**
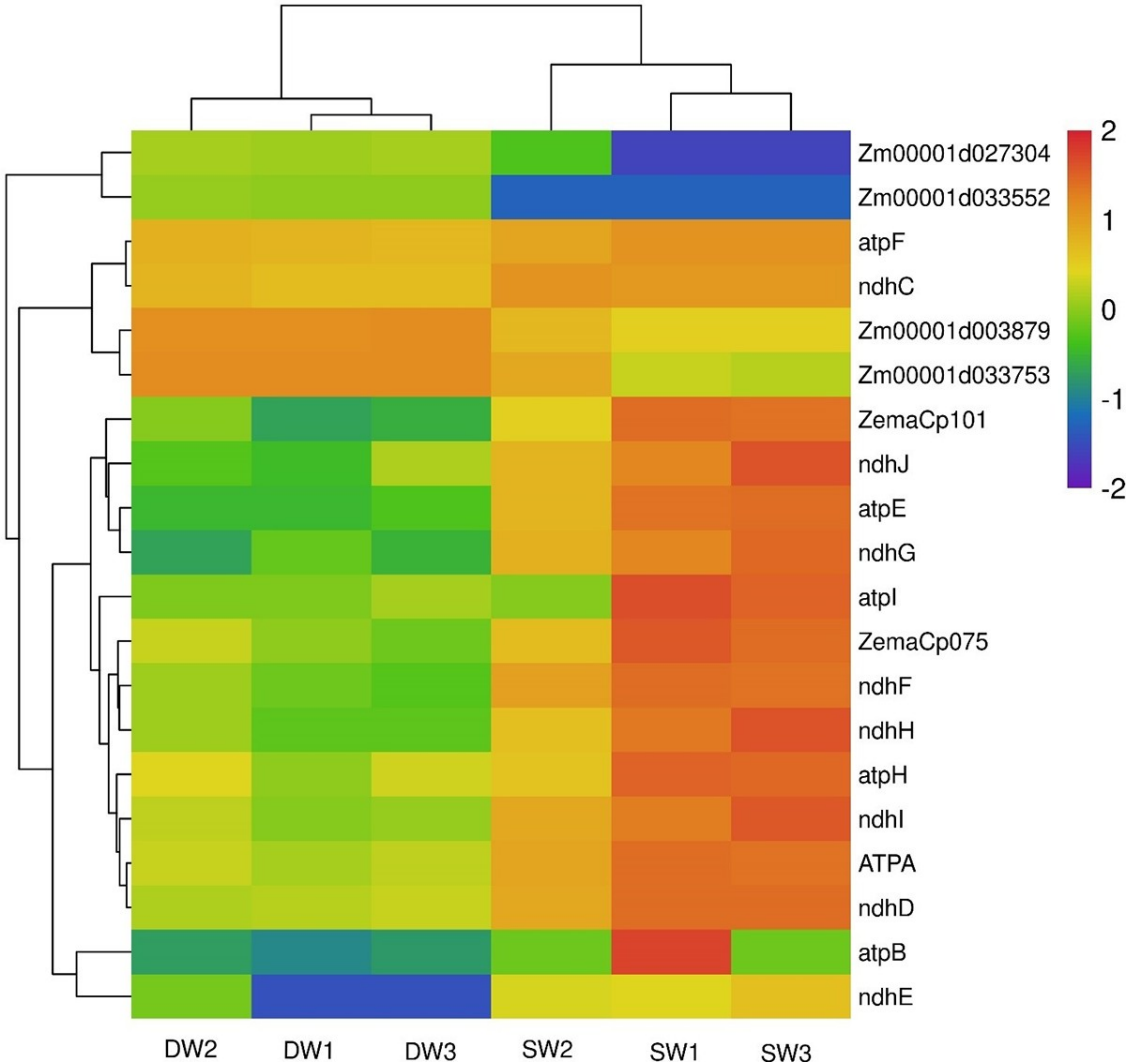
Heat map of oxidative phosphorylation pathway gene expression. SW refers to stable soil moisture content treatment, DW refers to alternately dry and wet treatment, and the number after that indicates the sample number.

The differential gene expression in photosynthesis pathway were as shown in Fig 5. Compared with DW treatment, 23 genes were up-regulated in maize under SW treatment. In the oxidative phosphorylation pathway, 6 genes were down-regulated and 14 genes are up-regulated (Fig 6). In order to determine the possible molecular mechanism of the corn response to soil water content, we determined the criteria of FC>100, | log_2_ (FC) |>6 and P<0.001 for differently expressed gene selection. There were 11 highly expressed genes under negative pressure irrigation (S1 Table). Photosystem II protein D1 (*PsbA*) and photosystem II protein D2 (*PsbD*) were up-regulated by 256.17 and 382.91 times respectively in maize under SW treatment (S3 Fig); *PsbC*, *PsbB*, *PsbE* and *PsbF* were up-regulated by 76.07, 120.02, 520.29 and 2944.02 times, respectively and other 17 genes including *PsbL* were significantly up-regulated. In Photosystem I system, *PsaB* and *PsaI* were up-regulated by 127.62 and 82.78 times, respectively and *atpB* (EC: 7.1.2.2) was up-regulated by 192.29 times. In the Oxidative phosphorylation pathway (S2 Table), the high-expressed genes satisfying FC>100, | log2 (FC) |>6 and P<0.001 were *atpE*, *atpB*, *ndhE* and *ndhG* high-fold up-regulated genes.

### Validation of gene expression profiles by RT-PCR

The RT-PCR was used to verify the differentially expressed genes revealed by RNA-seq. Expression trends of differentially expressed genes were consistent in both methods (S5 Fig). The top six significantly regulated genes were in photosynthesis pathway and involved two GO terms of chloroplast thylakoid membrane (*psaB*, *psbD*) and plastid (*psbA*, *psbF*, *ndhC*, *ndhG*).

## Discussion

The use of artificial irrigation to keep soil water content stable and within the range of suitable crop growth has a good effect on improving the efficiency of irrigation water use [17]. It has always been a challenge to keep the soil water content stable in the range suitable for crop growth. However, researchers have invented the NPI irrigation system, which achieved to maintain soil water content relatively stable [18]. Although the soil moisture content of SW has certain fluctuations during the growing season, it was always maintained within the range of 80±2% of the field water holding capacity. This range was within the design error range to ensure the success of this experiment.

Under adversity conditions, plants will produce a large amount of reactive oxygen species (ROS) which can lead to plant death in severe cases [19]. In the long-term evolution process, plants have formed a ROS removal system to remove excessive ROS [20]. The three most important enzymes for removing ROS in plants are SOD, POD and CAT. Under harsh conditions, the activities of these enzymes is increased. Therefore, the changes in antioxidant enzyme activities can be used as important indicators of crops in adversity conditions [21]. According to the results of this experiment, the activities of SOD and CAT of plants under SW treatment were significantly lower than those of plants under DW treatment (Table 2).This is related to the balance of active oxygen metabolism of maize under stable soil water content [22]. Free proline, soluble protein and soluble sugar are important small molecules related to plants stress and their content will be increased significantly when crops grow under adversity conditions [23]. In this study, the contents of the three osmotic adjustment substances in plants under SW treatment were lower than those under DW treatment which indicates that plants under DW conditions were subjected to more stress compared with plants under SW conditions. This may be the possible reason why the photosynthetic rate and nitrate reductase activity of plants under SW treatment were significantly higher than that of plants under DW treatment.

Stable and suitable soil water content can promote the growth of plant [24], thus significantly increase the accumulation of dry matter in the plant. In this study, the accumulation of dry matter in plants under SW conditions was significantly higher than that of plants under DW conditions. Accumulation of dry matter is the basis for the formation of corn yield and the increase in accumulation of dry matter is conducive to the increase of the final yield of maize [25,26]. The increase in accumulation of dry matter is directly related to the amount of photosynthetic products [27]. In this study, it was found that under SW conditions the rate of photosynthesis was increased (Table 3), increasing accumulated dry matter content in maize. Therefore, the increased photosynthetic capacity of maize under stable soil water content is the direct cause of the increase in yield [28,29].

Under appropriate stable soil water content, crop yields significantly increases [13]. The potential of utilization of crop yield is directly related to the increase in dry matter accumulation and the improvement of economic coefficient [15]. In this study, the economic coefficient of plants under SW treatment increased in 2019 and 2020 (Table 4). This is because the yield of maize increased hence the potential of maize production also increased [15]. Crop economic coefficient is affected by many factors. In this experiment, the increase of maize economic coefficient under stable soil water content related to coordination between vegetative growth and reproductive growth [13,30]. Stabilizing soil water content increased corn yield and EWUE hence achieving the goal of saving water and increasing production [9,31].

Under the condition of stable soil water content, there was no water stress during the growth period, which reduces the activity of antioxidant enzymes and the content of osmotic adjustment substances. This promoted the leaf growth and the relative content of chlorophyll [13,32]. The rate of photosynthesis in maize was significantly increased, which increased the accumulation of photosynthetic products, dry matter, yield and economic water use efficiency [18,33]. Lack of compensating growth effect of maize under the stable soil water content, enhances coordination of vegetative and reproductive growth, thereby increasing the economic coefficient, and hence promote maize production [24].

It was found that the number of down-regulated genes in corn leaves was greater than that of up-regulated genes under contents conditions controlled by NPI. Overall, the high-fold down-regulation of related genes in protein targeting chloroplast GO term may be one of the reasons for the decrease in chlorophyll content. Further, the high-fold down-regulation of oxidoreductase activity GO term related genes may be an important reason for the reduction of corn antioxidant enzyme activity and content of osmotic regulators.

Through the GO function enrichment analysis of differential genes, it was found that more different genes belong to the GO terms of chloroplast, plastid and thylakoid. The results of this study showed that the chloroplasts, plastids, thylakoids and other cellular components directly related to photosynthesis of maize would change significantly under the stable soil water content controlled by NPI. This is also a possible reason for the increase of photosynthetic rate, dry matter accumulation and yield of maize [34].

Through KEGG pathway analysis of differentially expressed genes, we also found that differentially expressed genes were significantly enriched in photosynthesis related pathways, such as photosynthesis, oxidative phosphorylation and ribosome pathway. In the pathway related to photosynthesis, 11 highly up-regulated genes were found. The four genes encoding structural proteins of photosystems, including *PsbE*, *PsbF*, *PsbA* and *PsbD*. These genes were also directly related to photosynthesis and were significantly up-regulated. The expression of genes encoding structural proteins of photosystems are positively correlated with photosynthetic capacity [35–37]. This is also the reason why the photosynthetic rate of maize significantly increases under stable soil water content controlled by NPI. In addition, *atpB*, which is directly related to the formation of ATP, was significantly up-regulated. This helps the corns to convert light energy into ATP for plant use and hence may be genes directly related to the increase in corn yield [34]. Therefore, it is deduced that the enhancement of photosynthetic capacity and energy metabolism of maize is the main physiological and molecular mechanism in response to stable soil water content.

## Conclusion

This study analyzed the physiological and molecular mechanism of yield increasing in maize under different soil water content conditions. The results showed that the increase in dry matter accumulation and yield was directly correlated with soil moisture during plant growth period. Transcriptome analysis revealed that there were 3397 differentially expressed genes between DW and SW treatments and among which 1165 genes were up-regulated whereas 2232 were down-regulated. These differentially expressed genes were mainly enriched in photosynthesis and oxidative phosphorylation pathways. In photosynthesis pathway, eleven genes including *PsbE*, *PsbF*, *PsbA*, *PsbD*, proteins encoding PSII and *atpB* encoding ATP synthase CF1 beta subunit were highly up-regulated. Eighteen genes of oxidative phosphorylation pathway were also up-regulated. These include the genes such as *atpE* and *atpB*, encoding ATP synthase and *ndhE* and *ndhG* encoding NADH dehydrogenase. These up-regulated genes may play an important role in response of corn to stable soil water content for increase in corn yields.

## Supporting information

S1 Fig A. PCA analysis diagram of maize leaf gene differences; B is the volcano map of gene expression.
(DOCX)

S2 Fig GO Functional classification of differentially expressed genes in maize leaves. (DOCX)

S3 Fig The effect of stabilizing soil water content on the photosynthesis pathway of maize. (DOCX)

S4 Fig The effect of stabilizing soil water content on the oxidative phosphorylation pathway of maize. (DOCX)

S5 Fig RT-PCR validation of differential transcription identified by RNA-seq. (DOCX)

S1 Table Photosynthesis pathway highly upregulated genes. (DOCX)

S2 Table Oxidative phosphorylation pathway highly upregulated genes. (DOCX)

S3 Table Primer synthesis information. (DOCX)

S1 File (TXT)

S2 File (TXT)

S3 File (FASTA)

S4 File (TXT)

S5 File (FASTA)

S6 File (FASTA)

S7 File (FASTA)

S8 File (FASTA)

S9 File (TXT)

S10 File (FASTA)
