# Supplementary material for: Transcriptome analysis reveals the molecular mechanism of yield increases in maize under stable soil water supply
Source: PLoS One. 2021 Sep 24;16(9):e0257756. doi: 10.1371/journal.pone.0257756 (PMC8462687; doi:10.1371/journal.pone.0257756)
Supplement: S1 Fig — A. PCA analysis diagram of maize leaf gene differences; B is the volcano map of gene expression. (DOCX) [file pone.0257756.s001.docx]

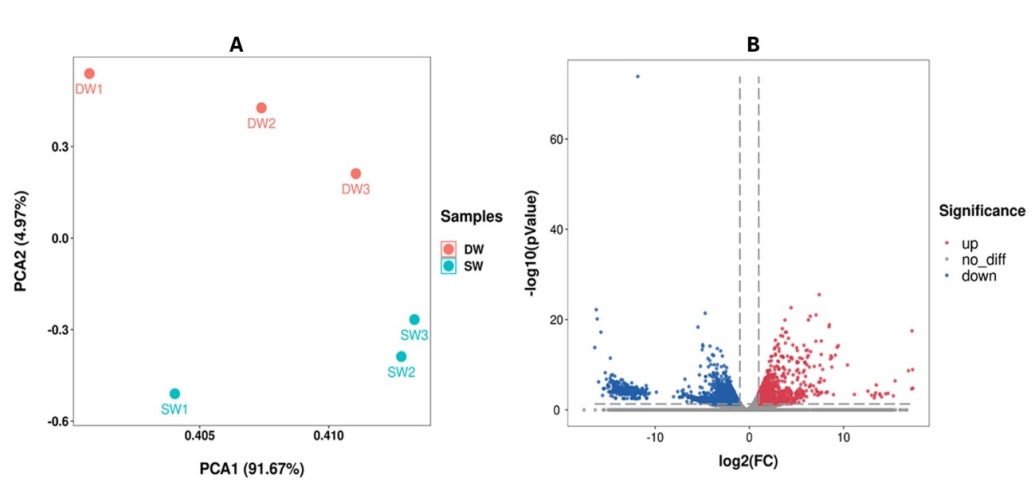


Fig. S1 A. PCA analysis diagram of maize leaf gene differences; B is the volcano map of gene expression, blue is the down-regulated gene, red is the up-regulated gene.
