# Supplementary figures and images for: Transcriptome analysis reveals the molecular mechanism of yield increases in maize under stable soil water supply
Source: PLoS One. 2021 Sep 24;16(9):e0257756. doi: 10.1371/journal.pone.0257756 (PMC8462687; doi:10.1371/journal.pone.0257756)

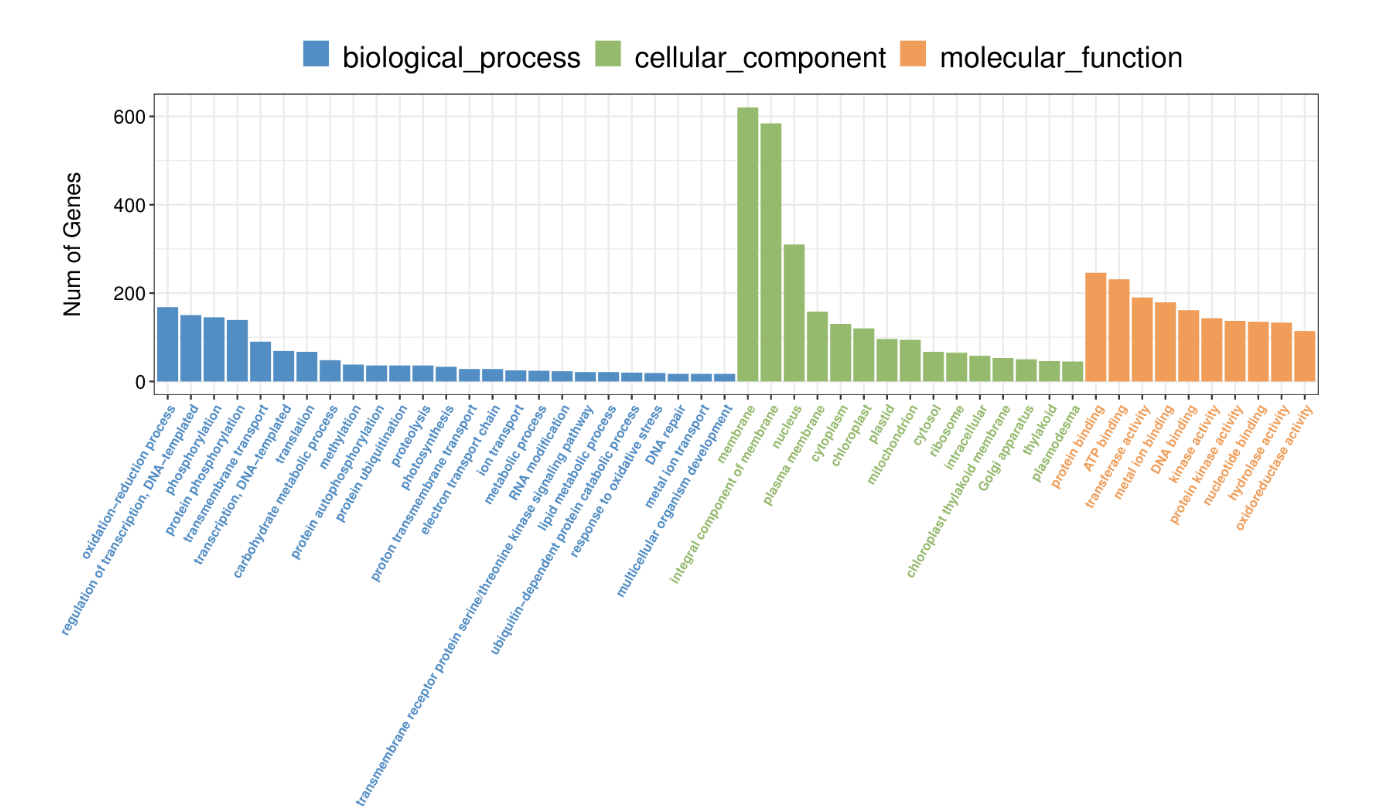


Fig. S2 GO Functional classification of differentially expressed genes in maize leaves

Supplement: S2 Fig — (DOCX) [file pone.0257756.s002.docx]
