# Supplementary material for: Transcriptome analysis reveals the molecular mechanism of yield increases in maize under stable soil water supply
Source: PLoS One. 2021 Sep 24;16(9):e0257756. doi: 10.1371/journal.pone.0257756 (PMC8462687; doi:10.1371/journal.pone.0257756)
Supplement: S3 Fig — (DOCX) [file pone.0257756.s003.docx]

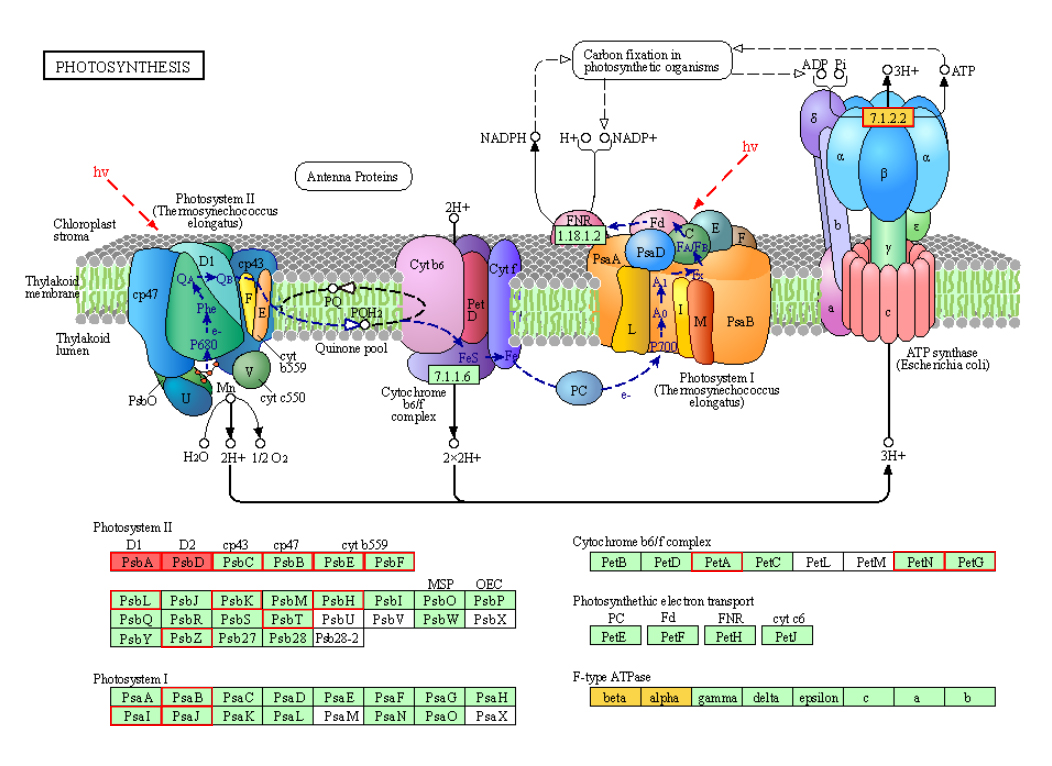


Fig. S3 The effect of stabilizing soil water content on the photosynthesis pathway of maize. Red boxes indicate up-regulated genes.
