# Supplementary material for: Transcriptome analysis reveals the molecular mechanism of yield increases in maize under stable soil water supply
Source: PLoS One. 2021 Sep 24;16(9):e0257756. doi: 10.1371/journal.pone.0257756 (PMC8462687; doi:10.1371/journal.pone.0257756)
Supplement: S4 Fig — (DOCX) [file pone.0257756.s004.docx]

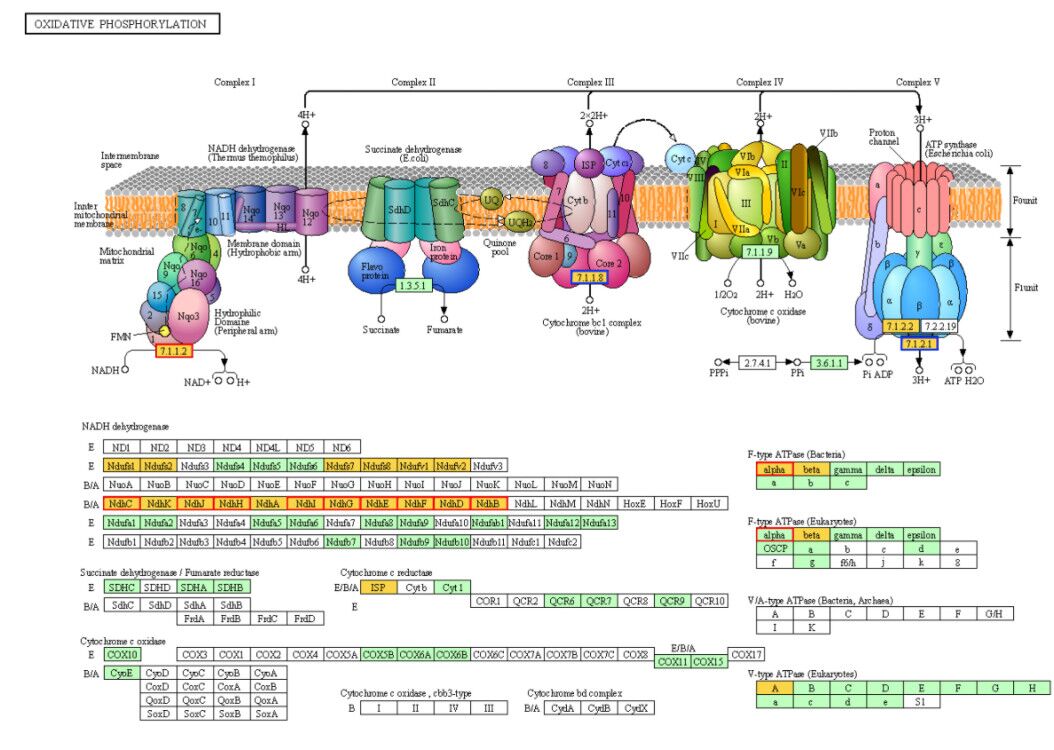


Fig. S4 The effect of stabilizing soil water content on the Oxidative phosphorylation pathway of maize. The red box indicates the up-regulated gene, and the blue indicates the down-regulated gene.
