# Supplementary material for: Transcriptome analysis reveals the molecular mechanism of yield increases in maize under stable soil water supply
Source: PLoS One. 2021 Sep 24;16(9):e0257756. doi: 10.1371/journal.pone.0257756 (PMC8462687; doi:10.1371/journal.pone.0257756)
Supplement: S5 Fig — (DOCX) [file pone.0257756.s005.docx]

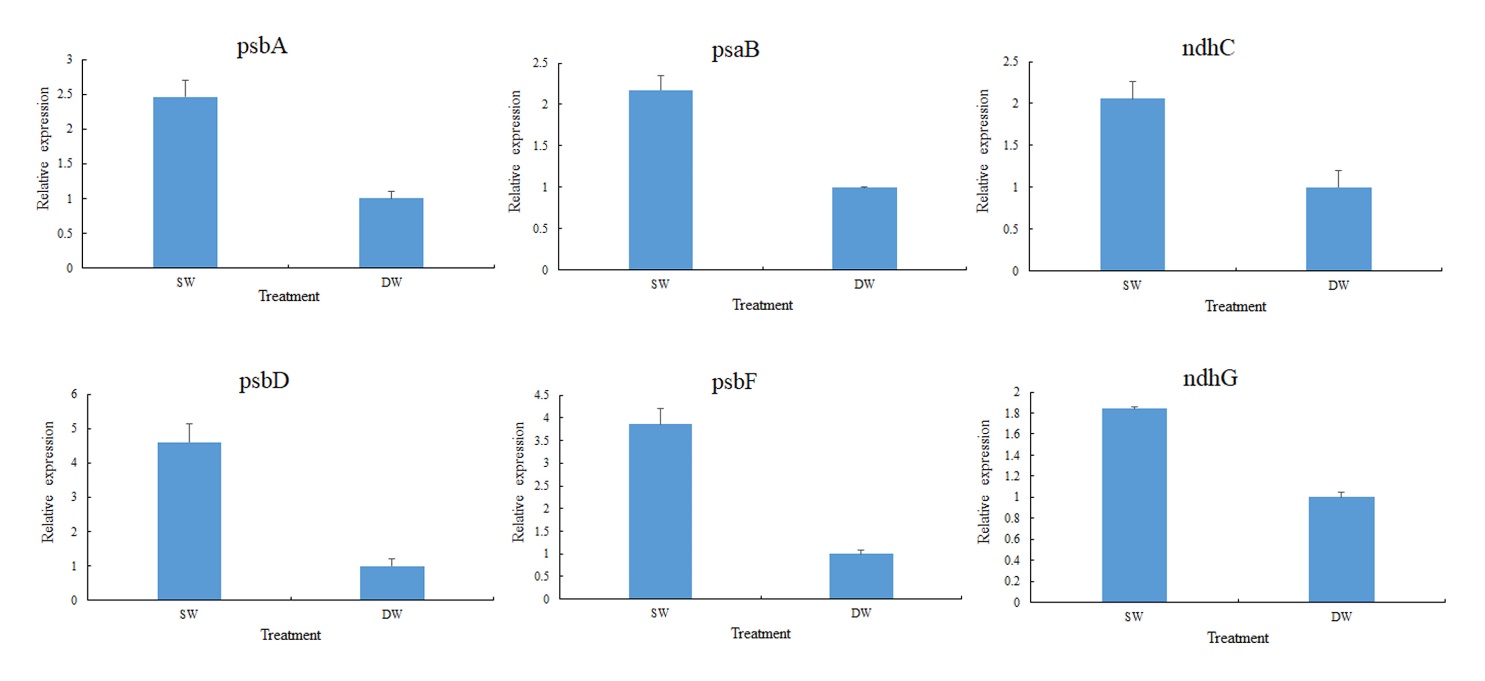


Fig. S5 qRT-PCR validation of differential transcription identified by RNA-Seq. SW is treatment for stabilizing soil moisture content, DW is treatment for alternating dry and wet.
