# Supplementary material for: Transcriptome analysis reveals the molecular mechanism of yield increases in maize under stable soil water supply
Source: PLoS One. 2021 Sep 24;16(9):e0257756. doi: 10.1371/journal.pone.0257756 (PMC8462687; doi:10.1371/journal.pone.0257756)
Supplement: S1 Table — (DOCX) [file pone.0257756.s006.docx]

Table S1 Photosynthesis pathway Highly upregulated genes

| Gene_ID | Gene_name | pvalue | FC | log2(FC) |
| --- | --- | --- | --- | --- |
| ZemaCp002 | psbA | 0 | 256.17 | 8.00 |
| ZemaCp007 | psbD | 0 | 382.91 | 8.58 |
| ZemaCp022 | psaB | 0 | 127.62 | 7.00 |
| ZemaCp030 | atpE | 0 | 138.60 | 7.11 |
| ZemaCp031 | atpB | 0 | 192.29 | 7.59 |
| ZemaCp038 | psbL | 0 | 127.05 | 6.99 |
| ZemaCp039 | psbF | 0 | 2944.02 | 11.52 |
| ZemaCp040 | psbE | 0 | 520.29 | 9.02 |
| ZemaCp048 | psbB | 0 | 120.02 | 6.91 |
| ZemaCp088 | ndhE | 0 | 163.88 | 7.36 |
| ZemaCp089 | ndhG | 0 | 185.11 | 7.53 |
