# Supplementary material for: Transcriptome analysis reveals the molecular mechanism of yield increases in maize under stable soil water supply
Source: PLoS One. 2021 Sep 24;16(9):e0257756. doi: 10.1371/journal.pone.0257756 (PMC8462687; doi:10.1371/journal.pone.0257756)
Supplement: S2 Table — (DOCX) [file pone.0257756.s007.docx]

Table S2 Oxidative phosphorylation pathway Highly upregulated genes

| Gene_ID | Gene_name | pvalue | FC | log2(FC) |
| --- | --- | --- | --- | --- |
| ZemaCp030 | atpE | 0 | 138.60 | 7.11 |
| ZemaCp031 | atpB | 0 | 192.29 | 7.59 |
| ZemaCp088 | ndhE | 0 | 163.88 | 7.36 |
| ZemaCp089 | ndhG | 0 | 185.11 | 7.53 |
