# Supplementary material for: Transcriptome analysis reveals the molecular mechanism of yield increases in maize under stable soil water supply
Source: PLoS One. 2021 Sep 24;16(9):e0257756. doi: 10.1371/journal.pone.0257756 (PMC8462687; doi:10.1371/journal.pone.0257756)
Supplement: S3 Table — (DOCX) [file pone.0257756.s008.docx]

Table S3 Primer synthesis information

| Gene number | Primer name | sequence (5'to3') | Annealing temperatureTM |
| --- | --- | --- | --- |
| 1 | 18S-F | CGCTCCTACCGATTGAAT | 60 |
|  | 18S-R | GATAAGGTTCAGTGGACTTCT |  |
| 2 | ZemaCp029-F | GTTAGTGAAGGACCAGAGAAG | 60 |
|  | ZemaCp029-R | CGTATTCGGAATTGTAACCAAG |  |
| 3 | ZemaCp089-F | TGTCGCACAACTTCTTATTTATG | 60 |
|  | ZemaCp089-R | CCATCTCCAATAGTCCAATAATTC |  |
| 4 | ZemaCp022-F | ACGGACACCTTTGGCTAA | 60 |
|  | ZemaCp022-R | GGCTAATCCGACCAATCTTG |  |
| 5 | ZemaCp002-F | TATCATCAACCGTGCTAATCT | 60 |
|  | ZemaCp002-R | CCATTAAGGTAAGGAACTTCAAG |  |
| 6 | ZemaCp007-F | AGTGCTATTGGCGTAGTT | 60 |
|  | ZemaCp007-R | TGGTGTAGAAAGTCTCAAATTC |  |
| 7 | ZemaCp039-F | GACCATAGATCGAACCTATCCTA | 60 |
|  | ZemaCp039-R | TTGCTGATATTGATCCCAAGAAA |  |

Table S3 Raw data NCBI public database storage information

| accession | study | object_status | bioproject_accession | biosample_accession | sample_name | library_ID | title | filename | filename2 | filename3 |
| --- | --- | --- | --- | --- | --- | --- | --- | --- | --- | --- |
| SRR14120312 | SRP312939 | new | PRJNA718001 | SAMN18520659 | DW_leaf1 | Library 1 | DW_leaf1 | DW_leaf1_Clean_Data1.fq | DW_leaf1_Clean_Data2.fq |  |
| SRR14120311 | SRP312939 | new | PRJNA718001 | SAMN18520660 | DW_leaf2 | Library 2 | DW_leaf2 | DW_leaf2_Clean_Data1.fq | DW_leaf2_Clean_Data2.fq |  |
| SRR14120310 | SRP312939 | new | PRJNA718001 | SAMN18520661 | DW_leaf3 | Library 3 | DW_leaf3 | DW_leaf3_Clean_Data1.fq | DW_leaf3_Clean_Data2.fq |  |
| SRR14120309 | SRP312939 | new | PRJNA718001 | SAMN18520662 | SW_leaf1 | Library 4 | SW_leaf1 | SW_leaf1_Clean_Data1.fq | SW_leaf1_Clean_Data2.fq |  |
| SRR14120308 | SRP312939 | new | PRJNA718001 | SAMN18520663 | SW_leaf2 | Library 5 | SW_leaf2 | SW_leaf2_Clean_Data1.fq | SW_leaf2_Clean_Data2.fq |  |
| SRR14120307 | SRP312939 | new | PRJNA718001 | SAMN18520664 | SW_leaf3 | Library 6 | SW_leaf3 | SW_leaf3_Clean_Data1.fq | SW_leaf3_Clean_Data2.fq |  |
